# The Profiles and Antecedents of Supervisor-Directed Emotional Labor Strategies: The Role of Self-Identity and LMX Orientations in Emotional Labor Strategy

**DOI:** 10.3390/bs13100865

**Published:** 2023-10-22

**Authors:** Ranran Wang, Sang-Joon Kim, Insu Kwon

**Affiliations:** 1 Department of Business Administration, Sejong University, Seoul 05006, Republic of Korea; wangrnr@gmail.com; 2 Department of Business Administration, Ewha Womans University, Seoul 03760, Republic of Korea; s.kim@ewha.ac.kr

**Keywords:** supervisor-directed emotional labor, self-identity level, LMX orientations, latent profile analysis

## Abstract

This study has two purposes. The first is to determine whether subordinates employ alternative combinations of emotion regulation strategies toward their supervisors beyond merely using surface and deep labor from the person-centered perspective. The second purpose is to understand why such acts of emotion regulation occur in interactions between employers and employees in the typical workplace. Utilizing latent profile analysis on data from 232 office employees in Beijing, China, collected using a two-stage sampling technique, four distinct supervisor-directed emotional labor profiles (i.e., deep actors, non-actors, moderators, and regulators) are identified. We find that these profiles are differentiated by several factors (i.e., individual identity, relational identity, and LMX orientations). Moreover, our findings suggest that employees exhibiting high levels of relational identity are more predisposed to act as deep actors, whereas individuals with high levels of individual identity are prone to being regulators as opposed to becoming deep actors, non-actors, or moderators. In addition, our results also suggest that LMX orientations have moderating effects on the relationships between self-identities and supervisor-directed emotional labor strategies. Overall, the results of this study expand the potential dimensionality of supervisor-directed emotion regulation strategies (e.g., regulating and non-acting) and bridge a gap in our understanding of the factors impacting supervisor-directed emotional labor.

## 1. Introduction

Emotion regulations are inextricably linked to interpersonal interactions [1]. As supervisors play a significant role in shaping their subordinates’ performance, rewards, and professional growth [2], employees may employ emotion regulation strategies to align with their employers’ expectations. Consequently, it is crucial to comprehend how and why employees practice emotional labor toward their supervisors within their daily work routines.

While there has been a substantial examination of emotional labor strategies, much of the research has primarily concentrated on emotion regulation strategies within customer interactions in the service industry, particularly surface acting (SA) and deep acting (DA) [3,4,5]. The integrated application of these strategies within the context of daily supervisor–subordinate interactions, however, remains insufficiently explored. Although existing literature offers crucial insights into emotional labor strategies toward supervisors [6,7], positing that subordinates utilize these strategies to influence supervisors for desired outcomes, the understanding of why employees engage in diverse combinations of emotional labor strategies toward their supervisors is still limited. Thus, this research embarks on an in-depth exploration of these areas, aiming to provide a comprehensive understanding of the multiple applications and potential antecedents of emotional labor strategies in supervisor–subordinate interactions.

In the context of daily work, subordinates are unlikely to consistently express their genuine emotions to their supervisors. In certain situations, they may need to employ either surface acting (SA) or deep acting (DA) as the main up-influence strategies to effectively manage their relationship with supervisors during daily interactions [6]. Surface acting involves simply putting on the expected emotional mask and focusing on emotional responses (faking unfelt emotions and/or suppressing true emotions) [8]. Deep acting entails regulating one’s emotions from the depths of one’s heart and attempting to generate the feelings that must be conveyed [8,9]. However, due to the ongoing, frequent, and intricate nature of supervisor–subordinate interactions, employees may find themselves tasked with managing dynamically shifting emotions in their interactions with supervisors, even when not explicitly governed by prescribed display rules [10]. Therefore, employees might employ multiple emotional labor strategies contingent upon their individual objectives and circumstances. For instance, some subordinates may engage in both deep and surface acting to varying degrees within their emotional labor roles, while others may predominantly rely on one strategy over the other [10,11].

Recent studies on supervisor-directed emotional labor have indeed predominantly utilized a variable-centered approach to examine the relationships between each strategy (e.g., surface acting and deep acting) and outcomes, for instance, previous research on supervisor-directed emotional labor has revealed that deep acting toward supervisors can positively impact employees’ work outcomes, such as enhancing their relationships with their supervisors or fostering the perception of their competence by the supervisors. Conversely, surface acting is linked to adverse work outcomes for employees, such as low levels of supervisors’ liking or poorer relationships with the supervisors [6,7]. These studies have mainly focused on investigating the separate effects of surface and deep acting on work outcomes from a variable-centered perspective. However, this variable-centered approach does not fully capture the complex ways in which employees may use both surface and deep acting strategies to manage emotional labor strategies toward their supervisors. For example, some subordinates may heavily rely on both surface and deep acting, while others may predominantly use one strategy over the other. Additionally, although deep acting is theoretically considered more beneficial than surface acting, these benefits may not be realized when individuals display high levels of both surface and deep acting [11].

Addressing the perplexing results associated with deep acting, person-centered approaches can contribute to the clarification of potential theoretical inconsistencies found in the existing emotional labor literature, concentrating on personal profiles instead of specific associations between variables [12]. Person-centered approaches, which concentrate on identifying employee sub-groups characterized by distinct configurations or profiles across a set of variables, provide a more intuitive framework for assessing cumulative effects without presuming specific patterns such as linearity among these variables. The adoption of this approach enables us to gain insights into how various employees characteristically employ diverse combinations of emotion regulation strategies in their interactions with supervisors during their daily work routines. Then, using the person-centered approach, scholars can use the potential profiles as a means to examine the relationships between profiles and their antecedents and outcomes [10,11,13], decreasing the unobserved heterogeneity problems that are ignored in the prior studies that use the variable-centered approach [14].

Person-centered approaches have recently gained traction in investigating the amalgamation of emotional labor strategies within specific individuals operating in customer-oriented and coworker-oriented domains [10,11,13,15]. For instance, Gabriel et al. [11] employed a person-centered approach to discern five distinct emotional labor profiles within the customer service sector, namely surface actors, deep actors, regulators, low actors, and non-actors. Additionally, Gabriel et al. [10] identified four discrete categories of emotional labor strategies exhibited among colleagues, including deep actors, non-actors, low actors, and regulators. These studies demonstrated the potential for individuals to either exclusively adopt a single emotional strategy or simultaneously employ both strategies (surface acting and deep acting). Furthermore, despite the comprehensive consideration of a wide array of antecedents (e.g., positive/negative affectivity, display rules, emotion demand-abilities fit, prosocial motives, and impression management motives) for emotional labor profiles in related domains [10,11], a systematic exploration of predictors for these potential profiles in the context of supervisor-directed emotional labor remains pending.

Therefore, in this study, our initial objective is to assess whether subordinates employ various combinations of emotional labor strategies (e.g., surface acting, deep acting, regulating, non-acting, etc.) when interacting with their supervisors. Consistent with the approach employed in [10], we adopt a person-centered approach to identify subordinates who engage in distinctive forms of deep and/or surface acting when dealing with their supervisors. If, indeed, the profiles of supervisor-directed emotional labor exist, our subsequent research endeavor will revolve around the exploration of the determinants (e.g., individual identity, relational identity, and LMX orientations) influencing these distinct profiles.

## 2. Theory and Hypotheses

### 2.1. Supervisor-Directed Emotional Labor

Initially, the primary focus of emotional labor research was its implication in the service industry. Yet, in recent times, scholars have broadened their scrutiny to encompass emotion regulation in diverse workplace contexts beyond the service industry, such as interactions between supervisors and subordinates, among colleagues, and within teams [6,10,16]. However, previous supervisor-directed research has mainly focused on the consequences of emotional labor [6,7], and there is still a lack of research on the multiple combinations of supervisor-directed emotional labor strategies and the reasons why subordinates use different emotional labor strategies toward their supervisors. For example, how do employees use emotional labor strategies toward their supervisors? Would they use solely one strategy (e.g., surface acting or deep acting) or both two strategies toward their supervisors? And if different combinations of supervisor-directed emotional labor exist, what predictors will influence employees to select different emotional labor strategies toward their supervisors?

Past studies have explored the links between individual differences and surface acting (SA) and deep acting (DA), such as the big five personality traits, emotional expressivity, self-monitoring, and positive/negative affectivity [3,4,11,17]. More recently, studies have proposed that emotion regulation strategies can be classified as motivated behaviors [10,18], which encompass intrinsic or extrinsic motivation, prosocial motives, and impression management motives. For instance, Gabriel et al. [10] found that prosocial motivations are associated with adopting the deep acting strategy, whereas impression management motivations are linked to the regulating strategy (i.e., involving high levels of both surface acting and deep acting) during interactions with colleagues. As scholarly research progressively underscores motivation-centric factors within studies on emotion regulation [1], it becomes imperative to account for motivation-based individual variances in the decision-making procedures that underpin supervisor-directed emotional labor strategies.

Expanding on this foundation, the present study aims to examine if employees’ self-identity level, an essential self-regulatory mechanism [19,20], is related to employees’ choice of emotional labor strategies toward their supervisors. Drawing upon the Identity-Based Motivation Theory [20,21], individuals are inclined to act and interpret their experiences based on their self-identity foci. Consequently, self-identity can influence individuals’ motivations by generating distinct emotional display goals toward supervisors, depending on which level of identity is more salient [22]. However, at present, there is a lack of direct research examining how different levels of self-identity impact the selection of supervisor-directed emotional labor strategies. For instance, do individuals with a strong relational self-identity tend to engage in deep acting when interacting with their supervisors? Thus, in this study, we propose that chronic self-identity can act as a motivation-based variable that sheds light on individual differences among various supervisor-directed emotion regulation strategies.

Moreover, research has shown that employees engage in multiple exchange relationships to derive various benefits [23]. The multi-foci perspectives of social exchange [24,25,26] highlight that subordinates differentiate between their exchange relationships with supervisors, reciprocating accordingly towards the intended beneficiary. In other words, subordinates are likely to possess distinct motivations for emotional acting toward their supervisors based on their perceived LMX (e.g., social leader–member exchange and economic leader–member exchange) and then achieve their intended goals through these emotion regulation efforts. Notably, research has demonstrated that the interdependent self-concept has a positive association with LMX, as individuals with a robust interdependent self-concept tend to value and prioritize maintaining positive relationships and fulfilling role expectations [27]. Hence, it is essential to consider the potential interplay between contextual factors (e.g., SLMX and ELMX foci) and personal self-identity in determining how employees regulate their emotional labor strategies toward their supervisors. Therefore, this study also endeavors to explore whether subordinates’ LMX orientations serve as moderators in the relationship between self-identity levels and the profiles of emotional labor directed toward supervisors.

Thus, we first use the latent profile analysis to establish our supervisor-directed emotional labor actor profiles based on the two levels of use for surface and deep strategy, high use and low use, followed by past latent profile analysis studies [10,11]. Considering the more direct interest-based relationship between supervisors and subordinates than colleagues, subordinates may be hesitant to deliberately hide their fake and negative emotions to prevent upsetting their supervisors. Therefore, it is plausible that our study may also not represent the complete surface acting profile that is absent in previous studies [10,11]. In sum, as emphasized in our previous discourse on person-centered approaches, we anticipate identifying certain profiles, such as a profile characterized by high levels of deep acting and low levels of surface acting (deep actors), a profile distinguished by extremely low levels of deep and surface acting (non-actors), a profile exhibiting high levels of both deep and surface acting(regulators), a profile marked by comparably low levels of deep and surface acting (low actors), or a profile defined by similar moderate levels (moderators).

**Figure 1:**
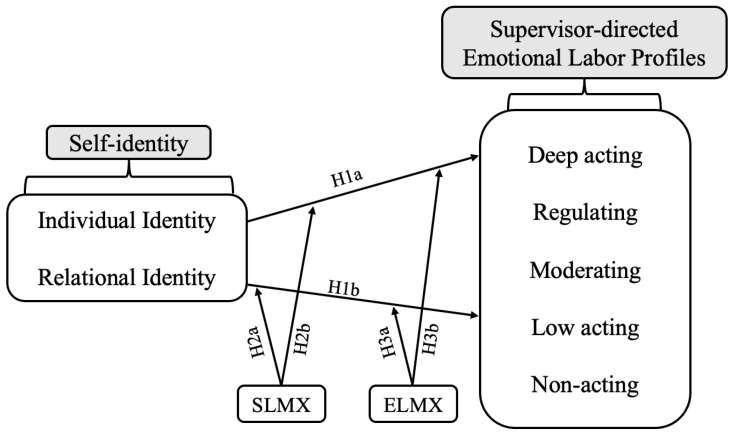
Oyserman2007SocialIdentityThe framework of research.

If the suggested potential profiles (e.g., deep actors, regulators, low actors, moderators, and non-actors) of employees engaging in supervisor-directed emotional labor indeed exist, our next step is to assess how diffident self-identities and Leader-Member Exchange (LMX) orientations predict the choice of distinct supervisor-directed emotional labor strategy profiles. The research model is depicted in Figure 1.

### 2.2. Self-Identity Level as a Predictor of Supervisor-Directed Emotional Labor Profiles

As a chronic representation of identity that promotes a self-definition anchored at the individual, relational, or collective level, the self-concept influences how people feel, think and behave [28]. Self-identity might have the potential to impact individuals’ emotion regulation motivations, leading to different emotional display objectives toward supervisors, depending on the more prominent level of self-identity.

At the individual level, individuals define themselves based on their sense of uniqueness and derive their self-worth from being different and superior to others [29]. Individuals at this level are driven by personal values and pursuits that maximize their self-interests, similar to individualism’s cultural value [30]. In work settings, employees with a strong sense of individual identity prioritize self-serving outcomes, such as pay and career development opportunities. Consequently, such employees are not predisposed to accruing social capital with group members and partners, nor do they receive the social support accompanying it. Employees with a strong sense of individual identity tend to be motivated by a concern for their advantage and well-being, with personal goals taking precedence [31]. At a minimum, individual identity is often characterized by traits of competitiveness, self-reliance, emotional detachment from in-groups, and hedonism [32]. In organizational settings, individuals with an individualistic orientation tend to behave in ways that help them acquire valued economic and socioemotional rewards and minimize the loss of investments they have already made. Therefore, employees with higher levels of individual identity may prioritize impressing their supervisors to maintain high levels of self-focus, which can manifest as instrumental behavior and a focus on personal pleasure.

Based on the interpersonal emotion regulation motivation (IERM) theory [1] and self-determination theory [33], motives that are higher in autonomy (e.g., coaching, instrumentality, compassion, hedonism) will be more likely to be pursued using deep acting interpersonal emotion regulation strategies, where the subordinates with high self-concern motivation may expend sustained effort to create an authentic change to the supervisors’ felt emotions (e.g., deep acting), while motives that are lower in autonomy, such as conformity and impression management, are more likely to be pursued through the use of surface acting strategies, where employees exert relatively less effort in the interaction process [1]. In addition, according to [34], individuals who are motivated by intrinsic hedonistic concerns are more likely to engage in deep acting and less likely to engage in surface acting, while those motivated by extrinsic organizational rules are more likely to engage in surface acting during customer service. Furthermore, Gabriel et al. [10] also argued that extrinsic motivations, such as impression management motives, make employees more likely to be regulators, utilizing both high levels of surface acting and deep acting when interacting with their colleagues.

Drawing on the aforementioned theoretical basis, we hypothesized that individuals possessing strong individual identities might exhibit emotion regulation toward their supervisors for two reasons. One reason might be driven by self-centered pleasure and hedonism, while the other might be motivated by self-interested impression management and instrumental gains. It is important to note that these employees’ concerns extend beyond purely instrumental interests, as they also attach significance to socioemotional outcomes like acknowledgment, esteem, and influence. In general, any incentive or punisher, tangible or otherwise, that has direct implications for the self is essential to employees with strong chronic individual identities.

Then, we postulate that employees who possess strong individual identities are unlikely to engage in surface acting simplistically and directly when confronted with the authority and power of their superiors. Instead, they may employ a combination of deep acting and surface acting to safeguard their own interests and gratify their instrumental and hedonistic motivations. Therefore, we hypothesize:

**H1a:**  *Employees with high levels of individual identities will be more inclined to be regulators compared to deep actors, moderators, low actors, or non-actors.*

Relational self-identity pertains to how individuals perceive themselves in relation to others who hold significance in their lives. At this level, individuals are driven by the welfare of the specific other, and appropriate role behaviors regarding a specific person determine self-worth [35]. Those with relational identities concentrate on connectedness with others [36]. This degree of self-identification underscores inter-individual relatedness, intimacy, and interdependency [37]. The fundamental motivation of relational self-identity lies in the dyad’s well-being, and self-esteem originates from the fulfillment of one’s role-relationship responsibilities.

In the work setting, people with high relational self-identities are focused on relationship development and maintenance, which are accomplished by internalizing the values and goals of their vertical and horizontal dyadic partners (supervisors and team coworkers). Moreover, based on regulatory focus frameworks, those high in relational self-identities generally aspire to advance their partners’ interests and maintain superior-quality relationships with them in the workplace [22]. Thus, we propose that those individuals exhibiting high levels of relational identities might employ deep acting toward their supervisors to demonstrate sincerity and friendliness, aiming to receive emotional reinforcement and social resources.

Moreover, Vos and van der Zee [38] have suggested that individuals who prioritize interpersonal relationships with others, such as those with relational identity orientations, are more likely to engage in prosocial behaviors toward their workgroup members. Previous research has also shown that individuals with high levels of agreeableness and trait-positive affectivity are more likely to engage in prosocial behaviors involving deep acting and genuine displays [17,39]. Furthermore, Gabriel et al. [10] posited that prosocial motives drove deep actors among coworkers. Based on the exchange theory, employees with high levels of relational identities are more likely to adhere to the norm of reciprocity and less likely to engage in rational and self-interested calculations, weighing the costs and benefits of their actions [40].

According to the presented theoretical framework, individuals with high levels of relational identities might be more likely to establish enduring and positive relationships and cultivate high-quality exchange relationships with their supervisors. Consequently, it is reasonable to propose that employees with high levels of relational identities might be more predisposed to selecting positive emotional responses and engaging in sincere emotion regulation toward their supervisors. Therefore, we hypothesize the following:

**H1b:**  *Individuals with high levels of relational identities will be more inclined to be deep actors compared to low actors, moderators, regulators, or non-actors.*

### 2.3. The Moderating Role of Lmx Orientations

LMX relationships comprise two distinct components: a social component (SLMX) and an economic component (ELMX), each characterized by different qualities [41]. Followers with different LMX orientations are likely to respond differently to relational self-identity and individual self-identity when choosing how to display emotions toward their supervisors. This is because high-quality leader–follower relationships may lead followers to integrate organizational norms into their self-conception, such as a relational or collective self-concept [42]. Hence, while employees may adopt different emotional labor strategies with their supervisors based on their levels of self-identity focus, the selection of these strategies might also be influenced by their perceptions of the exchange relationship with their supervisors. As a result, the emotion regulation direction of individuals towards their supervisors could be influenced by the various LMX relationship orientations, considering the initial emotion regulation choices made by individuals with distinct self-identities.

The SLMX relationship is characterized by a long-term orientation where each party expects some future return. Investment in the relationship is a crucial aspect closely linked with relationship-based trust [43] due to the inherent risk that the investment might not be reciprocated [44]. Moreover, in such relationships, the emphasis is on socio-emotional aspects such as “give and take” and “being taken care of” [45]. Lester et al. [46] argued that “social exchange involves expectations of reciprocity, which motivates self-interested and strategic exchanges”. In this context, followers with high SLMX orientations may seize the opportunity to act more genuinely to meet their supervisors’ emotional expectations and obtain their trust or related social resources. When individuals have high levels of relational identity, their self-worth is connected to their supervisor’s approval, as their esteem is augmented by constructing high-quality relationships and fulfilling their supervisor’s expectations. At the relational level, individuals are motivated to serve the well-being of their supervisors and fulfill their emotional needs and role obligations. Thus, we hypothesize that individuals with high levels of relational identity will be more likely to engage in sincere and friendly emotion regulation toward their supervisors (i.e., being deep actors) when they have high SLMX orientations, as it reinforces their original motivation derived from their relational identities.

Although individuals who prioritize their individual identities focus on self-interested rewards such as compensation or personal fulfillment from their work, those who possess high Social Leader–Member Exchange (SLMX) orientations may exhibit greater caution in evaluating the costs and benefits of their actions in a rational and long-term self-interested consideration. Moreover, employees with high SLMX orientations, aiming to establish enduring and mutually beneficial relationships with their supervisors, may strategically employ deep acting as an opportunistic approach to safeguard their interests. By acting more genuinely and positively towards their supervisors through deep acting, they seek to secure benefits such as being cared for and trusted. Hence, a strong SLMX orientation may potentially diminish the influence of self-interest-driven motives that stem from individual identity levels while enhancing motivations to fulfill their supervisors’ expectations when employees adopt emotional labor strategies towards their employers. In other words, high SLMX orientations may increase the motivation of subordinates with individual identities to use deep acting toward their supervisors and weaken their motivations to use superficial acting. Even in instances of employing deep acting in interactions with their supervisors, the individuals’ intentions could potentially remain strategic manipulation designed to safeguard their interests and enhance their personal growth. Accordingly, based on the theoretical and research findings presented earlier, it is reasonable to surmise that SLMX orientations may prompt individuals with individual identities to shift from their original role as regulators to deep actors. Therefore, we hypothesize the following:

**H2a:**  *SLMX orientation moderates the relationship between relational identity and supervisor-directed emotional labor strategies. Such SLMX orientation makes individuals with high levels of relational identities more inclined to become deep actors compared to other forms of actors (e.g., moderator, regulator, non-actor)*

**H2b:**  *SLMX orientation moderates the relationship between individual identity and supervisor-directed emotional labor strategies. Such SLMX orientation may prompt individuals possessing high levels of individual identity to transform from regulators into deep actors.*

While we propose that those possessing strong relational identities are more likely to engage in deep acting when interacting with their supervisors, and individuals with individual identities may employ both high levels of surface acting and deep acting as regulators, these choices could also be influenced by their Economic Leader-Member Exchange (ELMX) orientations. Contrasting with social exchange relationships, an economic exchange relationship tends to be more short-term oriented [45], encompassing minimal personal involvement and the exchange of tangible resources typically acquired through distinct quid pro quo transactions [47]. The ELMX orientation could intensify an individual’s instrumental motivation while maintaining relationships with their supervisors [48]. Song et al. [47] asserted that employees who emphasize their economic exchange relationship with an organization “are concerned with return equivalency, negotiate with their employer for rewards, lack patience or expectations for future returns, and ultimately prioritize self-interest”. Motivated by self-centered concerns, these individuals tend to concentrate on the direct benefits of their actions. Consequently, we hypothesize that higher ELMX orientations should neutralize the connections between relational identities and employees’ deep acting toward their supervisors. Because followers with higher ELMX orientations are less inclined to engage in long-term consideration and other interest-focused calculations of potential action outcomes, employees whose emotion regulation motivations are driven by ELMX orientations might be less focused on cultivating positive, long-term relationships and resource exchanges; instead, they are more preoccupied with their emotion regulation efforts yielding returns on their investments. Such instrumental concerns should align with surface acting, as this tactic may be adequate for attaining work-related objectives [34].

Though a more instrumentally driven ELMX relationship, motivated by immediate self-interest, may increase the likelihood of adopting surface acting behaviors towards supervisors, this potential could be constrained by the supervisors’ power. Followers with higher ELMX are more prone to engage in rational and self-interested calculations of potential costs associated with their direct surface actions toward their supervisors [40]. Hence, we speculated that individuals with high ELMX orientations might also use deep acting strategies toward their supervisors when their immediate self-interest is threatened, as they face the pressure of their supervisors’ authority. Such deep acting skills will likely be driven by instrumental motives [49], aiming to shield their interests from potential harm. In this case, employees with higher ELMX orientations are apt to strategically oscillate between high levels of deep acting and surface acting toward their supervisors (e.g., regulators) to avoid provoking their supervisors and negatively impacting their interests. Thus, we predict that employees with high relational identities and ELMX orientations may enact deep acting strategies paired with surface acting when interacting with their supervisors, depending on the situations and their self-interest protection. We also speculate that ELMX orientation will strengthen the possibility that individuals with individual identities to be regulators (i.e., using both high levels of surface acting and deep acting toward their supervisors). Accordingly, we hypothesize:

**H3a:**  *ELMX orientation moderates the relationship between relational identity and supervisor-directed emotional labor strategies. Such high ELMX orientation makes individuals with high levels of relational identities more inclined to change from deep actors to regulators.*

**H3b:**  *ELMX orientation moderates the relationship between individual identity and supervisor-directed emotional labor strategies. High ELMX orientation makes individuals with high levels of individual identities more inclined to become regulators.*

## 3. Method

### 3.1. Participants and Procedure

We collected our sample using survey data from an extensive data company in Beijing, China. The sample collection was completed in two stages. First, we used the data collection platform of the data survey company to recruit volunteers willing to participate in the survey. Second, we distributed the questionnaire to each successful applicant based on the email address provided by the applicant. We initially collected a total of 400 applicants. We eliminated 168 samples (N = 106) who did not fill in all the questions and individuals who did not correctly answer the obstacle questions (to test whether the sample answered the questions seriously) we set (N = 33). We also removed samples (N = 29) that did not correctly answer questions based on comparing positive and reversed. In the end, we left 232 pairs of valid sample data. Targeted participants were office employees (e.g., subordinates) from organizations in China that operate in service, retail, finance, telecommunications, medical institutions, manufacturing, real estate, education, government, etc. The sample includes 49.10% males and 50.90% females; the average age was 30.03 years. Education levels ranged from those receiving a high school diploma and lower to those receiving a Ph.D. Most employees have obtained a bachelor’s degree (72.4%). The employees’ mean working lifetime is 6.30 years. The mean tenure with the current supervisor was 3.94 years.

### 3.2. Measures

Most of the survey items were initially developed in English. We transferred the items into Chinese. Responses were given on a five-point scale ranging from 1 (strongly disagree) to 5 (strongly agree) unless otherwise stated.

**Supervisor-directed emotional labor.** We assessed surface acting using seven items (Cronbach’s alpha = 0.95) adapted from Diefendorff et al.’s (2005) study, making suitable adjustments to align with the specific traits of supervisor–subordinate interactions (e.g., “I put on a “mask” to deal with my supervisor in an appropriate way”). To evaluate deep acting (Cronbach’s alpha = 0.91), we employed an initial scale comprising three items derived from Grandey’s [5] deep acting scale (e.g., “I try to actually experience the emotions that I must show to my supervisor”) and four items adapted from Kruml and Geddes’ [50] emotive effort scale (e.g., “I try to change my actual feelings to match those that I must express to my supervisor”). We altered the referent of the items by substituting ’customers’ with ’supervisor’ to gauge the extent to which subordinates engaged in surface acting and deep acting directed at their leader.

**SLMX and ELMX.** We measured social and economic LMX employing the scales from Dysvik et al. [48]. A sample item for the measurement of ELMX (Cronbach’s alpha = 0.90) is “I watch very carefully what I get from my immediate supervisor, relative to what I contribute”, whereas a sample item for the measurement of SLMX (Cronbach’s alpha = 0.90) is ”My relationship with my immediate manager is based on mutual trust”.

**Self-identity.** We assessed self-identity using the Levels of Self-Concept Scale [51], which consists of three five-item scales. A sample item measuring individual self-identity (Cronbach’s alpha = 0.75) is “I often compete with my friends”. In contrast, a sample item measuring relational self-identity (Cronbach’s alpha = 0.82) is “I value friends who are caring, empathic individuals”.

### 3.3. Analytic Approach

In this study, we first conducted reliability and Exploratory Factor Analysis (EFA) to verify the reliability and statistical validity of the measured variables (i.e., surface acting, deep acting, relational identity, individual identity, SLMX, and ELMX) designed in our research. The value of Kaiser–Meyer–Olkin (KMO) (0.905) and Bartlett’s Test ($\chi^{2}=4357.197$, df=325, p<0.001) demonstrated that our dataset is appropriate for factor analysis. Following the recommendations of Podsakoff et al. [52], we examined the severity of common method bias (CMB) in our study. We conducted Harman’s single-factor test on all items. Analysis results indicated that the cumulative total majority is 75.135%, and the total variance of the first component is 33.194%. Furthermore, we also performed confirmatory factor analysis (CFA) on a six-factor model comprising surface acting, deep acting, relational identity, individual identity, SLMX, and ELMX. The model fit the data well ($\chi^{2}=142.26,\;df=107,\;\chi^{2}/df=1.33,\;p<0.001,\;GFI=0.94,\;AGFI=0.91$, CFI=0.99,RMSEA=0.04) fit the data properly. Overall, these results signify that no significant risk of CMB exists in our research.

In the second step, we executed latent profile analysis (LPA) in Mplus 8.00 [53]. Then, the LPA was conducted as previously outlined to determine the number of profiles fitting the data (e.g., profile enumeration). Scholars [54] recommend profile solutions with (a) lower AIC, BIC, C-AIC, and SSA-BIC values; (b) significant LMR and BLRT statistics (*p* < 0.05); and (c) higher Entropy values. Simulations suggest that BIC, C-AIC, and SSA-BIC are more optimal in identifying the best solution [55]. However, BIC and C-AIC statistics tend to underestimate the number of profiles, while SSA-BIC and BLRT statistics overestimate. Thus, we evaluated the theoretical implications of the profiles to ensure parsimony (e.g., where extracted profiles in a solution are not theoretically redundant). In the third step, we assessed the antecedents of supervisor-directed emotion labor profiles using the R3STEP command in Mplus [56]. This step was conducted in MPLUS. Finally, to test interaction terms, multinominal logistic regression analyses were performed to evaluate the interaction effect between self-identities (i.e., individual identity, relational identity) and LMX orientations (i.e., SLMX, ELMX) on supervisor-directed emotion labor strategies. This step was conducted in SPSS.

## 4. Results and Discussion

Table 1 presents all variables’ means, standard deviations, and bivariate correlations. The mean for surface emotional labor was 2.68, and for deep emotional labor was 3.58. The mean for individual identity was 3.70, and that for relational identity was 4.36. The mean for ELMX was 2.87, and that for SLMX was 3.60. As the results are shown in the correlation matrix, which indicates provisional support for all “main effect” hypotheses dealing with self-identities and emotional labor types, relational identity and SLMX are positively related to deep emotional labor (r = 0.319, *p* < 0.01); (r = 0.454, *p* < 0.01), relational identity negatively related to surface emotional labor (r = −0.149, *p* < 0.05); Interestingly, ELMX is positively correlated with both surface emotional labor (r = 0.677, *p* < 0.01) and deep labor (r = 0.377, *p* < 0.01), which paves the way for our further in-depth analysis of why this phenomenon occurs.

Table 2 presents fit Statistics for potential latent profile configurations. A 4-profile solution was selected due to its lower LL, AIC, BIC, and SSA-BIC values and significant LMR and BLRT values compared to the two- and three-profile solutions. This choice was made for three reasons. Firstly, the four-profile solution displayed decreased C-AIC and BIC values relative to the two- and three-profile solutions. Secondly, the BLRT and LMR statistics were significant. Although the five-profile or larger solutions had marginally reduced LL, AIC, and SSA-BIC metrics compared to the five-profile solution, the LMR statistic was non-significant, and the additional extracted profiles did not provide considerable theoretical advancements. Consequently, the four-profile solution was maintained. These findings substantiated the decision to preserve the 4-profile solution within this research.

Table 3 provides descriptive details for each latent profile identified in the 4-profile solution. Specifically, we discovered deep actors (21.55% of the sample), who displayed relatively higher levels of deep acting (M = 3.92) alongside low levels of surface acting (M = 1.64); non-actors (23.28% of the sample), characterized by negligible to low levels of surface acting (M = 1.68) and deep acting (M = 2.25); regulators (34.48% of the sample), typified by high levels of surface (M = 4.01) and deep acting (M = 4.15), who demonstrated high aptitude in both emotional strategies; and moderators (moderate surface acting (M = 2.73) and slightly higher deep acting (M = 3.74), categorized by displaying intermediate levels of surface and deep emotional labor strategies compared to the other three profiles. This profile represented 20.69% of the participants. This naming approach aligns with Fouquereau et al.’s [13] examination of emotional labor profiles, where the researchers likewise identified a moderator profile characterized by moderate surface acting levels and comparatively heightened deep acting.

These findings suggest that distinct supervisor-directed emotional labor actors exist in the typical workplace. These results coincide and diverge from the profiles previously identified in customer and coworker interactions [10,11] in meaningful ways. For instance, similar to Gabriel et al.’s [10] study, the absence of surface actors—characterized by high surface acting and low deep acting—identified in Gabriel et al.’s [11] employee–customer interactions study is noteworthy. Unlike employee–customer interactions, which may be fleeting, the long-term nature of subordinate–subordinate interactions and the specificity of the subordinate–subordinate relationship could encourage subordinates to develop different emotion regulation mechanisms towards their supervisors. When faced with their supervisors’ power and authority, subordinates might be less inclined to engage in surface acting directly to safeguard their own interests. Furthermore, indifferent to the findings of Gabriel et al.’s [10] study, the data suggested that levels of deep acting toward supervisors are relatively higher than those in coworker interactions, leading to isolating a group of moderators than purely low actors in coworker interactions. This difference could prompt inquiries about supervisor-subordinate relational specificity and class hierarchy concerns.

Regarding antecedents (Table 4), the findings imply a strong association between deep acting and relational identity level. The result demonstrated that a one-unit increase in relational identity level heightened the probability that individuals belonged to the deep actor profile over the regulator (OR = 8.17), non-actor (OR = 0.14), or moderator (OR = 0.14) profiles. Consequently, Hypothesis H1a is supported.

Regarding individual identity levels, we anticipated that employees with individual identity levels would be more likely to be regulators compared to other profiles. The results showed that a one-unit increase in individual identity level heightened the probability that individuals belonged to the deep actor profile over non-actor (OR = 0.31) or moderator (OR = 0.44) profiles. A one-unit increase in individual identity level also increased the probability that individuals belonged to the regulator profile over the non-actor (OR = 0.12) or moderator (OR = 0.23). Based on the findings, employees with high individual identity levels typically belong to the two categories of deep actors and regulators. However, there was a slightly statistically significant differentiation in this antecedent between regulators and deep actors. Specifically, we also observed that individual identity made employees more likely to be regulators compared to deep actors (OR = 0.53), suggesting that individual identity level is more likely to motivate employees to regulate emotional displays to their supervisors by using both high levels of surface acting and deep acting versus using solely deep acting, or not engaging in such regulatory efforts towards supervisors. Thus, Hypothesis H1b is supported. Together, these results provide insight into hypotheses 1a and 1b, demonstrating that the theoretical antecedents of self-identity levels differentiate profiles of supervisor-directed emotional labor actors.

Table 5 displays the outcomes of the interaction effects of the SLMX and ELMX orientations. Moderator analysis revealed that SLMX orientation significantly moderated the relationship between relational identity and emotional labor strategies. This is evidenced by a significant interaction term among participants who are deep actors compared to other profiles. SLMX orientation will cause individuals with high relational identity more inclined to be deep actors, supporting Hypothesis H2a (*p* < 0.05). Confirming the moderating effect of SLMX orientation on the relationship between individual identity and emotional labor strategies, the results are not significant. Then, Hypothesis H2b is not supported.

To further examine the interaction effects of ELMX, as summarized in Table 5. Initially, individuals with high levels of relational identity with their supervisors belonged to the profile of deep actors. However, when the individuals have high ELMX orientations, they are inclined to change from deep actors to regulators or non-actors toward their supervisors (OR = 1.79, *p* < 0.1); (OR = 2.51, *p* < 0.01), partially supporting Hypothesis H3a. Moreover, the result also indicates that ELMX orientation can cause people with individual identities to be more inclined to become regulators or non-actors than deep actors (OR = 1.96, *p* < 0.01; OR = 1.74, *p* < 0.05). Although there is a slightly statistically significant difference between the deep actor profiles and the regulator profiles initially, the data indicated that a high level of ELMX orientation could enhance the motivation of employees with individual identities to become regulators compared to deep actors. Additionally, ELMX orientation makes employees with high levels of individual identity more inclined to become regulators when the moderator profile is compared with the regulator profile (OR = 0.41, *p* < 0.01), partially supporting Hypothesis H3b.

Our findings substantiated that subordinates’ emotion regulation towards their supervisors can fluctuate based on their LMX orientations. Significantly, the LMX orientations can interact with employees’ self-identity levels, ultimately impacting their strategic expression of emotions toward their supervisors. For instance, assuming the subordinates aspire to establish a long-term, mutually beneficial social exchange relationship with their supervisors, the SLMX orientation will prompt individuals with relational identities to be more predisposed toward engaging in deep acting. Conversely, if employees aim to establish short-term economic exchange relationships with their supervisors, the ELMX orientation will counterbalance the subordinates’ natural inclination to engage in only deep acting towards their supervisors. They may need to combine this approach with surface acting or reduce their emotional regulating behaviors toward their supervisors, which may result in them becoming regulators or non-actors. Thus, this study reveals that the orientation of the exchange relationship with supervisors is also a crucial factor influencing the selection of emotional labor strategies directed toward supervisors.

## 5. General Discussion

Driven by Gabriel et al.’s [11] finding that employees report employing multiple emotion regulation strategies in the customer service domain, the primary goal of this research is to examine how and why emotion regulation strategies are integrated by subordinates in the common workplace domain to manage their emotion displays toward their supervisors from the person-centered perspective. Indeed, prior research has mostly overlooked this issue.

We discovered that supervisor-directed emotion regulation strategies are combined into distinct emotion regulation styles using a latent profile analysis. Specifically, we identify four profiles: deep actors, regulators, moderators, and non-actors. Furthermore, these profiles were associated with different levels of self-identities (i.e., relational identity and individual identity). Deep actors appear to be motivated by relational identity, whereas regulators are motivated by individual identity.

This study also extends its inquiry to the under-researched area of the influences of Leader–Member Exchange (LMX) orientations on employee–employer exchanges. While there is evidence available on the outcomes of employee–organization exchange relationships [58], less is known about the moderating influences of LMX orientations on ongoing employee–employer exchanges. Thus, the current study examines the moderating role of LMX (SLMX and ELMX) orientations in the relationship between supervisor-directed emotional labor strategies and self-identities. Mainly, our findings reveal that high-level ELMX orientations prompt individuals with high levels of relational identity to transition between emotion regulation profiles, for example, high-level ELMX orientations will make individuals with high levels of relational identity more likely to act toward their supervisors, from deep actors to regulators or non-actors, and make individuals with individual identities more inclined to do regulating acting toward their supervisors. Moreover, SLMX orientation bolsters the inclination for relational identity individuals to perform deep acting toward their supervisors.

These findings offer an exploration into the relationship between emotion regulation strategies and LMX orientations, enhancing understanding of their combined impact on self-identities and supervisor-directed emotion regulation strategies. By shedding light on how LMX orientations guide individuals with distinct self-identities in emotion regulation, this study enriches LMX literature, augments our grasp of workplace behaviors and motivations, and serves as a cornerstone for subsequent LMX-focused endeavors.

## 6. Theoretical Implications

Our research provides several valuable theoretical contributions to advancing emotional labor and social exchange theory literature. We broaden the understanding of supervisor-directed emotional labor strategy literature from a person-centered perspective. Our study provides a new view on the question of how subordinates perform emotional labor strategies toward their supervisors. Although researchers have started to address this issue by emphasizing the benefits of supervisor-directed emotion regulation in ordinary organizational life, most studies have concentrated on the two main quantitative dimensions of emotion regulation toward the supervisor [6,7], namely deep acting and surface acting, which overlooked the process of potential changes in person-centered emotion regulation in specific situations. In line with the prior study on emotion regulation among colleagues [10], we identified more potential supervisor-directed emotion regulation profiles through a human-centered perspective, such as regulators, moderators, and non-actors.

Moreover, our study fills a gap in prior research on why employees are staged differently in emotional labor strategies toward their supervisors. Drawing on identity-based motivation theory [59], we add new insights into how different self-identity levels affect the subordinates’ motivation to select emotional displays toward their supervisors. This study complements the motivational elements of the emotion regulation literature by delineating a direct connection between levels of self-identity and emotional labor strategies. By exploring employees’ motives for regulating their emotions toward their supervisors, this study provides insight into the reasons behind the different combinations of supervisor-directed emotional labor that employees utilize. It is likely that self-identity plays a critical role in the selection process of supervisor-directed emotional labor strategies, similar to its importance for leadership [19], organizational justice [22], and commitment and motivation at work [35].

Furthermore, our study contributes to the literature on social exchange theory and emotion regulation literature by examining how LMX orientations moderate the relationship between self-identity levels and employees’ emotion regulation strategies toward their supervisors. By examining the role of LMX orientations in how different LMX relationships’ motives toward supervisors affect employees’ emotion regulation behaviors, we shed light on the situational variables that have been overlooked in the literature on emotional labor toward supervisors. This expands the current understanding of LMX theory and emotional labor research and underscores the importance of considering contextual factors in examining emotion regulation behaviors in the workplace.

## 7. Practical Implications

In addition to its theoretical contributions, our research offers practical insights for organizations and managers. We found that supervisor-directed emotional labor is prevalent as an up-influence tool in common workplaces. Our findings highlight the importance of understanding the underlying motivations behind subordinates’ emotion regulation strategies. This insight can help managers better comprehend and address the motivations driving their subordinates’ emotion regulation behaviors, which in turn can foster healthier supervisor–subordinate relationships.

By exploring the interaction effects between self-identity levels and LMX orientations, our research provides managers with a new perspective for understanding the emotion regulation behaviors of their subordinates. For example, recognizing that employees with relational identity and social exchange relational motivation are more likely to be deep actors can help managers identify and support those who may be more invested in building meaningful relationships within the workplace.

In conclusion, our study has significant implications for understanding emotional labor strategies and supervisor–subordinate relationships, emphasizing that emotional labor behaviors are influenced not only by personal effort but also by a combination of individual and situational factors. By applying these insights, organizations and managers can better understand and support their employees, foster more effective supervisor–subordinate relationships, and ultimately improve overall workplace well-being and productivity.

## 8. Limitations and Future Directions

While our study has provided valuable insights into supervisor-directed emotional labor and its implications, it is important to acknowledge the limitations and contemplate potential directions for future research. First, the data collected in this study was self-reported and obtained from a single source, which may have limitations in terms of biases and subjectivity. Future research could employ multi-source data or use observational methods to complement self-report measures.

Second, our study was conducted in China, and its findings may not generalize to other cultural contexts. Future research should examine the relationships between emotional labor strategies, self-identity, and LMX orientations across different cultural settings to provide a more comprehensive understanding of these phenomena.

Third, since employees might hide their true feelings toward supervisors, future research should develop more refined measures to distinguish emotion display targets and explore the reasons behind using different emotional strategies. Future research could explore additional antecedents (e.g., traits, motives, emotional intelligence, etc.) and work environment-related factors (e.g., negative events, interaction characteristics, social information gating, etc.) that may influence these strategies.

Finally, our study captured general emotion regulation strategy tendencies but did not account for the dynamic nature of emotional labor. Future research should adopt a dynamic perspective on emotion regulation towards supervisors and explore how employees’ strategies change over time or in response to specific events.

## Figures and Tables

**Table 1:** Mean (M), standard deviation (S.D.), and correlation.

	Mean	SD	1	2	3	4	5	6	7	8	9
1. Subordinate Gender	1.51	0.50	−								
2. Subordinate Age	30.03	6.71	0.183 **	−							
3. Working Tenure with current Supervisor	3.94	2.90	0.144 *	0.691 **	−						
4. Relational Identity	4.36	0.57	0.11	0.10	0.05	−					
5. Individual Identity	3.70	0.73	−0.01	−0.145 *	−0.13	0.12	−				
6. SLMX	3.60	0.81	−0.05	0.00	0.01	0.334 **	0.323 **	−			
7. ELMX	2.87	1.09	−0.10	−0.167 *	−0.174 **	−0.142 *	0.320 **	−0.02	−		
8. Surface Emotional Labor	2.68	1.10	−0.11	−0.213 **	−0.254 **	−0.149 *	0.259 **	−0.01	0.677 **	−	
9. Deep Emotional Labor	3.58	0.92	−0.10	−0.03	−0.07	0.319 **	0.357 **	0.454 **	0.377 **	0.506 **	−

Note: N=232; *. Correlation is significant at the 0.05 level (2-tailed). **. Correlation is significant at the 0.01 level (two-tailed).

**Table 2:** Latent profile enumeration fit statistics.

Model	Log Liklihood	AIC	BIC	SABIC	Entropy	Smallest Class %	LMR *p*-Value	LMR Meaning	BLRT *p*-Value
1	−3956.376	7956.751	8032.579	7962.851	−	−	−	−	−
2	−3277.300	6622.599	6739.788	6632.026	0.968	0.423	<0.001	2 > 1	<0.001
3	−3053.487	6198.973	6357.523	6211.728	0.947	0.211	0.0062	3 > 2	<0.001
4	−2947.982	6011.964	6211.875	6028.045	0.946	0.207	0.0036	**4 > 3**	<0.001
5	−2873.751	5887.503	6128.774	5906.911	0.938	0.125	0.0881	5 <4	<0.001

Note: N=232; LL = log-likelihood; AIC = Akaike information criteria; BIC = Bayesian information criteria; SSA-BIC = sample-size adjusted BIC; LMR = Lo, Mendell, and Rubin [57] test; BLRT = bootstrapped log-likelihood ratio test.

**Table 3:** Descriptive information by latent profile.

	% of Sample	Surface Acting	Deep Acting
Deep Actor	21.55%	1.64	3.92
Non-Actor	23.28%	1.68	2.25
Regulator	34.48%	4.01	4.15
Moderator	20.69%	2.73	3.74

Note: N=232; values represent the mean level of surface acting and deep acting in each latent profile.

**Table 4:** Antecedents (R3STEP) results surrounding self-identity level and emotional labor strategies.

Profile Comparisions																		
Antecedents	Non-Actor v. Moderator			Non-Actor v. Deep Actor			Non-Actor v. Regulator			Moderator v. Deep Actor			Moderator v. Regulator			Deep Actor v. Regulator		
	Coef.	SE	OR	Coef.	SE	OR	Coef.	SE	OR	Coef.	SE	OR	Coef.	SE	OR	Coef.	SE	OR
Individual Identity	−0.34	0.26	0.71	−1.17 ***	0.33	0.31	−1.79 ***	0.39	0.12	−0.82 **	0.27	0.44	−1.45 ***	0.35	0.23	−0.63 ${}^{†}$	0.34	0.53
Relational Identity	−0.06	0.33	0.94	−2.00 ***	0.54	0.14	0.10	0.33	1.11	−1.94 ***	0.56	0.14	0.16	0.32	1.18	2.10 ***	0.53	8.17

Note: N=232; OR = odds ratio; all values are estimates from the R3STEP logistic regression analyses. Positive values indicate that higher values on the antecedent make a person more likely to be in the first latent profile out of the two being compared; negative values indicate that higher values on the antecedent make a person more likely to be in the second latent profile. We took the absolute value of the logistic regression coefficients to calculate the odds ratio; positive and negative values are interchangeable in this analysis and are only used to reflect the direction of the comparison being made. ${}^{†}\;p<0.1$; ** *p* < 0.01; *** *p* < 0.001.

**Table 5:** Multinomial Logistic regression on interaction effect of self-identity, SLMX, and ELMX.

Profile Comparisions															
Antecedents	Non-Actor v. Deep Actor			Moderator v. Deep Actor			Deep Actor v. Regulator			Non-Actor v. Regulator			Moderator v. Regulator		
	Coef.	SE	OR	Coef.	SE	OR	Coef.	SE	OR	Coef.	SE	OR	Coef.	SE	OR
Individual Identity	−1.17 ***	0.33	0.31	−0.82 **	0.27	0.44	−0.63 ${}^{†}$	0.34	0.53	−1.79 ***	0.39	0.12	−1.45 ***	0.35	0.23
Relational Identity	−2.00 ***	0.54	0.14	−1.94 ***	0.56	0.14	2.10 ***	0.53	8.17	0.10	0.33	1.11	0.16	0.32	1.18
II × SLMX	−	−	−	−	−	−	−	−	−	−	−	−	−	−	−
RI × SLMX	−0.88 **	0.30	0.41	−0.96 **	0.29	0.38	0.73 **	0.27	0.48	−0.15	0.21	0.86	−0.23	0.22	0.80
II × ELMX	0.55 *	0.26	1.74	−0.22	0.30	0.80	−0.67 **	0.24	1.96	−0.12	0.20	0.89	−0.89 **	0.27	0.41
RI × ELMX	0.92 **	0.32	2.51	0.37	0.33	1.45	−0.58 ${}^{†}$	0.31	1.79	0.34 ${}^{†}$	0.21	1.41	−0.21	0.27	0.81

Note: N=232; ${}^{†}\;p<0.1$; * *p* < 0.05; ** *p* < 0.01; *** *p* < 0.001.

## Data Availability

The data of this study are available upon reasonable request.
